# Eye Contact Is a Two-Way Street: Arousal Is Elicited by the Sending and Receiving of Eye Gaze Information

**DOI:** 10.3389/fpsyg.2019.01262

**Published:** 2019-06-04

**Authors:** Michelle Jarick, Renee Bencic

**Affiliations:** Atypical Perception Laboratory, Department of Psychology, MacEwan University, Edmonton, AB, Canada

**Keywords:** gaze perception, eye contact, skin conductance, social interaction, eye contact effect

## Abstract

Research shows that arousal is significantly enhanced while participants make eye contact with a live person compared to viewing a picture of direct or averted gaze. Recent research has pointed toward the potential for social interaction as a possible driving force behind the arousal enhancement. That is, eye gaze is not only a signal perceived but also a signal sent out in order to communicate with others. This study aimed to test this by having dyads engage in eye contact and averted gaze naturally, while wearing sunglasses, and while blindfolded; such that the gaze signals were clear, degraded, and blocked, respectively. Autonomic nervous system arousal was measured *via* skin conductance response and level. The results showed that dyads exhibited the highest degree of arousal (increased skin conductance) while making eye contact (send/receive) compared to send-only or receive-only gaze trials; however, this was only the case if eye contact was clear. Once gaze information became degraded (by sunglasses or blindfold), arousal significantly decreased and was no longer modulated by the sending and receiving of gaze. Therefore, the arousal enhancement observed during eye contact is not only caused by receiving gaze signals (the focus of previous research) and should be more accurately attributed to the subtle interplay between sending and receiving gaze signals.

## Introduction

Eye gaze is a rich source of social information. Much research has shown that gaze direction is particularly useful to understand where someone’s attention is focused (e.g., Friesen and Kingstone, 1998; Hietanen, 1999; Frischen et al., 2007 for a review), to predict someone’s behavior and respond accordingly (e.g., avoiding someone walking toward you; Pelphrey et al., 2004), and most importantly, to know when the lines of communication are open or closed (e.g., Cary, 1978; Ho et al., 2015, for a review Risko et al., 2012; Canigueral and Hamilton, 2019). Indeed, humans have evolved to have eyes that are easily tracked, where our dark pupil is centered on a white sclera (Kobayashi and Kohshima, 1997). This high contrast between the pupil and sclera makes it easy for others to decipher in which direction the eyes are moving. In fact, research has shown that humans are as accurate as 1° of visual angle in determining others’ eye movements (Anderson et al., 2011). The high contrast of the eyes also attracts attention due to the complexity of the information the eyes portray. This attention-capturing effect of gaze has been observed from birth, with neonates preferring direct gaze over averted gaze of their mothers (Farroni et al., 2002; Senju and Csibra, 2008). In adults, looking at a photo of a person with direct gaze results in quicker processing of the face, as demonstrated by faster face detection (Senju et al., 2005) and facial gender discrimination accuracy (Macrae et al., 2002), compared to photos of averted gaze.

Recently, the literature on eye gaze has been more focused on face-to-face eye gaze, rather than traditional photos/videos presented in the laboratory, which have revealed some important differences (for reviews, see Risko et al., 2012; Canigueral and Hamilton, 2019). For instance, previous evidence suggests that when instructed to look at an image of a human face, participants visually attend more to the face (Castelhano et al., 2007), and particularly to the eyes of the social stimulus (Pelphrey et al., 2002; Birmingham et al., 2008, 2009) than to other features in the image. However, Laidlaw et al. (2011) tracked participants’ eyes while they were seated in a waiting room with a live confederate or a video of the confederate and found that participants fixated on the live confederate fewer times and for shorter durations relative to the video of the confederate. Thus, when the eyes were able to look back, participants’ gaze behavior changed. This difference in looking behavior between images and real-world contexts has also been supported in studies that measured autonomic nervous system arousal. For instance, research measuring skin conductance response (SCR) while participants viewed static images of faces with direct and averted gaze has shown only slight changes in SCR between gaze directions (Kampe et al., 2003; Kylliäinen and Hietanen, 2006; Joseph et al., 2008). Yet, Hietanen and colleagues have found a significant enhancement in SCR magnitude, when participants are exposed to direct over averted gaze with a live confederate. For example, Hietanen et al. (2008) found an increase in SCR as well as subjective reports of heightened arousal in participants exposed to direct eye gaze compared to averted gaze. Importantly, Hietanen et al. (2008) compared this SCR effect between live and static stimuli and found that the increase in SCR was only observed during direct gaze with the live confederate and not when the confederate was presented as a static image (Hietanen et al., 2008).

Furthermore, engagement with a real person has been shown to elicit different brain responses compared to an image/video. In multiple studies, Hietanen and colleagues used electroencephalography (EEG) to measure brain activity during direct versus averted gaze with a live person or a photo of a person. In two studies, the researchers took measures of hemispheric asymmetry in the frontal brain regions and found direct gaze with a real person elicited more EEG activation in the left hemisphere indicative of approach motivation compared to averted gaze that elicited more rightward hemispheric activation indicative of avoidance motivation (Hietanen et al., 2008; Pönkänen et al., 2011). Notably, these patterns of hemispheric activation were not observed for eye gaze with a person in a photo. In another study, Pönkänen et al. (2011) found enhanced face/eye-selective event-related brain wave (N170) to be significantly enhanced to direct gaze compared to averted gaze or closed eyes, but only when viewed from a live person. Similar differences between real people and images have been shown using functional magnetic resonance imaging (fMRI). Cavallo et al. (2015) had participants lie down in an MRI scanner while making direct or averted gaze with a photo of a person, a real person in the room (through a mirror), or with themselves in a mirror. They found that face-to-face gaze (direct and averted) elicited significant activation in brain areas involved in language comprehension and production (inferior frontal gyrus (IFG), premotor cortex, and supplementary motor area). Interestingly, the brain areas involved in inferring mental states during social interactions (anterior rostral medial prefrontal cortex or arMPFC) were only active when participants made direct gaze (eye contact) with a real person. Further analysis showed that there was an increase in connectivity between the IFG and arMPFC during live eye contact, suggesting that live gaze triggers a network of brain regions involved in the detection of communicative intentions and language.

Similar interpretations of real face-to-face interactions have been proposed. For example, Laidlaw et al. (2011) suggested that eye gaze with a live person opens up the possibility for interaction than when viewing someone in a video where interaction is not possible. In other words, a live person can look back at you and communicate social information that video stimuli are devoid of. Hietanen and colleagues have interpreted the attentional, physiological, and neurological differences between in direct gaze with a real person as reflecting the increase in self-awareness caused by being the focus of someone else’s gaze. This increase in self-awareness is proposed to encompass affective states, perception of another’s attention, self-referential processing, and reciprocal attention/interaction mechanisms (for a review, see Hietanen, 2018). As Gallagher (2014) put it, eye gaze in a “live encounter” is more than just a visual representation and encompasses the impact on the observers’ own system for action, which presents a “unique type of interaction.”

The notion of eye contact eliciting self-awareness was recently examined by Myllyneva and Hietanen (2015, 2016). The researchers measured changes in physiology [skin conductance response (SCR) and heart rate], brain waves (ERP; frontal P3 waveforms) as well as self-report measures of self-awareness while participants viewed another live person (model) behind a voltage-sensitive LC shutter. The visibility of the model was manipulated such that participants could: (1) clearly see the model and the model could see them, (2) believed the model could see them but they could not see the model, and (3) could not see each other. The key condition being the “belief” that someone could see them. The findings from Myllyneva and Hietanen (2015) showed a significant increase in SCR and P3 amplitude, as well as heart rate deceleration when participants “believed” the model could see them, but they could not see the model and self-awareness ratings were higher as well. These findings were replicated when the model wore sunglasses that either degraded eye gaze or blocked it completely (Experiment 2), where SCR increased when the eyes were visible and degraded but not when they were blocked. In Myllyneva and Hietanen (2016), the findings were contradictory. Those results revealed that self-awareness ratings were higher when participants could see the model or believed the model could see them, but physiological responses (skin conductance increase and heart rate deceleration) only differed when the participants could see the model. Thus, the “belief” that the model could see them was enough to increase the subjective experience of self-awareness, but not the objective physiological response associated with it. Myllyneva and Hietanen concluded that despite the experience of self-awareness, a social encounter must satisfy two conditions: (1) looking at another person and (2) being looked at by another person, to elicit physiological and neurological responses. However, the experience of self-awareness is a complex, high level, cognitive state, and likely encompasses many factors, which may have been represented differently between the two experiments conducted by Myllyneva and Hietanen.

We propose that a cleaner, low-level, perceptual explanation of gaze processing during live social interactions could be that people are simultaneously attending to two gaze signals: the gaze signal from others while at the same time monitoring their own gaze signals (i.e., being self-aware). While a video depicting social interaction only involves attending to the gaze signals coming from the person in the video. Furthermore, a live setting involves continuous, real-time monitoring, in which case the two sources of information could correlate or be independent depending on the context. Gobel et al. (2015) demonstrated the “sending” aspect of gaze behavior by having people filmed while watching videos of higher versus lower ranked individuals. The participants believed that their viewing behavior would later be watched by the individual in the video or that they would not be seen by anyone. When participants thought that their behavior was going to be later observed, they looked at the eyes less if the person was a higher ranked individual compared to lower ranked. Thus, those viewing higher ranked authoritative people were more controlled in their viewing behavior. In other words, participants were sensitive to the gaze signals they themselves were conveying when they believed someone might analyze them.

While mounting research has shown a definitive enhancement in physiological arousal when mutual eye gaze is made with a live person, it has yet to be shown whether arousal is being elicited by the eye gaze from others or the self-monitoring of our own gaze, or both. Here, we aimed to systematically examine the relationship between gaze signals and physiological arousal by manipulating the degree to which gaze signals are sent and received during a live social interaction. We measured the level of arousal of two strangers as they sat side-by-side on a couch and performed four different gaze “poses” (or trials) for 1 min each: (1) looked away from one another (*baseline/no-gaze trials*), (2) looked at their partner’s profile (*sent-only trials*), (3) had their partner look at them (*received-only trials*), and (4) made eye contact (*sent/received trials*). We then manipulated the clarity by which the gaze signals could be sent/received by either degrading them (one participant worse tinted sunglasses) or blocking them (one participant was blindfolded). We have three main hypotheses, one for each clarity condition. First, we believe that the significant enhancement of arousal observed in studies with live interactions is likely elicited by both *sending* gaze signals out (and the self-monitoring that goes along with that) as well as *receiving* gaze signals from others (and the interpretations that go along with that). Hence, our first prediction is that arousal will be enhanced the most when participants make eye contact because participants will be *sending* and *receiving* gaze signals, which has been already shown numerous times in previous research. We also predict that the sent-only and received-only trials will have a significant boost in arousal compared to the no-gaze trials, if arousal is associated with sending information. Note that there is already evidence that arousal is associated with receiving gaze from photos, but currently no research regarding sending gaze only. We also wanted to test whether the gaze signals need to be clear in order for those signals to be interpreted. For instance, situations do arise where the gaze signals are hard to receive even though eye contact is being made (e.g., imagine making eye contact with someone wearing sunglasses). Thus, our second prediction is that arousal during eye contact trials when gaze is degraded (with sunglasses) will be similar to the sent-only and received-only trials, where only one signal is influencing arousal. In the blindfolded condition, there are no signals sent or received and therefore, our third prediction is that the arousal during eye contact will not differ from the no-gaze trials. However, participants who are blindfolded are still aware that someone is looking at them and they are the focus of someone’s attention. Thus, if arousal is associated with the mental attribution of self-awareness (suggested by Myllyneva and Hietanen, 2015), then we might see a boost in arousal comparative to the sent-only trials.

There are few unique aspects of our design that warrant justification. To boost ecological validity, we had two participants perform the eye gaze trials together where previous studies typically used a participant and confederate. We chose not to use a confederate because we have observed in our previous (unpublished) studies that eye contact is something that people can quickly and easily habituate to. If confederates habituate to the gaze trials after one or two participants, then the eye gaze experience could be diminished for future participants, thereby giving them a different eye gaze experience. This habituation effect is also why we chose to only present each gaze trial (*away, sent-only, received-only, eye contact*) once per condition. In our most recent (unpublished) research, we have observed that participants arousal for eye contact becomes less and less the more times that they do it, and after three repetitions, arousal is no longer elicited to the same degree as it was in the first trial. Thus, we wanted to limit our trial number to three repetitions of each gaze trial. Lastly, we have an unusually long duration of eye contact (1 min) that is not typical of everyday eye contact that lasts only 3–5 s (Helminen et al., 2001). However, our question was not in relation to “making eye contact” *per se*, but rather associated with the *signals* sent and received, which would usually happen over multiple eye contact experiences during a conversation. Rather than having participants make eye contact naturally and unpredictably for a longer duration, we decided that we could encourage faster signal transfer if participants held eye contact for more than 5 s. While 1 min seems like a long duration, participants seem to be able to do it well and it allows us to get an idea of how arousal changes over time by evaluating skin conductance level, rather than just skin conductance responses. By taking advantage of this, we believe this to be the first study to show arousal as a function of time during social interactions.

## Experiment 1

### Method

#### Participants

Sixty-four MacEwan University undergraduate students (13 males, 47 females, mean age of 19 years old) were recruited in pairs (dyads) to participate in the study. There were 17 same-sex female dyads and 13 male-female opposite-sex dyads, all right-handed, and had normal or corrected-to-normal vision and hearing. Participants were compensated with 2% course credit toward their psychology course. All participants reported not knowing their partner, except for two same-sex dyads (*n* = 4) who indicated that they were friends and their data was excluded from the analysis. Experimental procedures were approved by the MacEwan Research Ethics Board. All participants gave informed, written consent prior to participation.

#### Materials and Procedure

Upon arrival to the laboratory, participant dyads were greeted by a female investigator and asked to first sit next to one another on the same couch. Tape was used to indicate the desired physical proximity between participants on the couch (this distance was approximately 30 cm apart). The investigator sat in front of both participants at a distance of 115 cm behind a table with a laptop computer. Participants were then fitted with physiological monitoring equipment (*Thought Technology, Inc.*), whereby two Ag/AgCl electrodes were attached to the palmar surface and of the distal phalanxes of their ring and index finger of their left hand. Their skin conductance level (SCL) was collected at a sampling rate of 256 samples per second. Participants were informed that their nervous system arousal would be monitored during different gaze trials and to try and remain as still as possible to prevent movement artifacts. Participants were also to try and stay as neutral as possible by keeping a neutral facial expression and withholding laughter or talking. All participants were able to remain fairly neutral with ease. Designated rest periods were inserted as 1–2 min breaks between each of the gaze trials, where participants could move, talk, or laugh during those breaks if need be. However, the investigator noted that participants did not talk much with each other during these breaks, but they would occasionally smile or laugh at the investigator.

**Figure 1:**
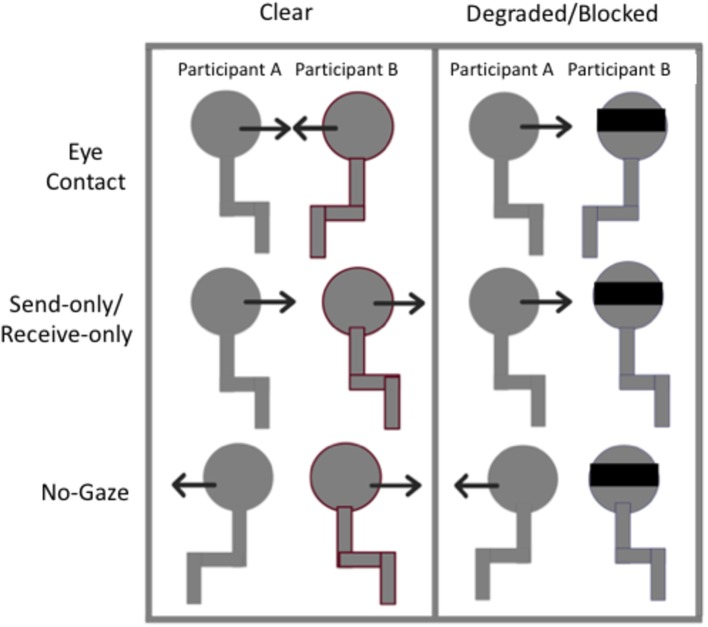
Schematic representation of the experimental set-up. In the Clear condition, participants performed the gaze trials (*no-gaze, send/receive, eye contact*) as one normally would. In the degraded and blocked conditions, participant B wore sunglasses or a blindfold, respectively.

The experiment was conducted in three blocks, one for each condition: clear (gaze was clearly observed), degraded (gaze was degraded by sunglasses), and blocked (gaze was blocked by a blindfold). The order of the blocks was counterbalanced in an ABC, BCA, CAB for each dyad. See Figure 1 for a schematic representation of the three conditions. For the *clear* condition, participants performed the gaze trials normally without any obstruction of gaze information. Both participants A (on the left) and B (on the right) could send and receive gaze information clearly. For the *degraded* condition, participant B was asked to wear sunglasses while performing the gaze trials. Note that in this condition, participant A would not be able to send or receive gaze information to participant B very well, since participant B’s gaze would be degraded by the tint of the sunglasses. Participant B, however, could send and receive gaze information from participant A just fine. For the *blocked* condition, participant B wore a blindfold while performing the gaze trials. In this condition, participants A and B could no longer send or receive gaze information with each other. However, it is important to note that the participants were still instructed on which gaze trial they were to complete, so when asked to make eye contact, for example, they would still turn their heads toward each other as if to make eye contact. Thus, the participant with the blindfold knew that there was someone looking at them during those trials.

Each block consisted of four gaze trials (*no-gaze, send-only, receive-only*, and *send/receive or eye contact*), where participants performed four “poses” for 1 min each: (1) *no-gaze* - participants looked away from each other by turning their head in the opposite direction from their partner, (2) *send-only trials*—participant looked at their partner’s profile by turning their head toward their partner, (3) *receive-only trials*—participant was looked at by their partner while looking straight ahead and, (4) *eye contact trials*—both participants engaged in eye contact by only turning their head toward one another. Participants began and ended each trial on a verbal command from the investigator (i.e., “ready, set, go” and “stop”), who kept track of the 1-min interval and event-marked the SCL recording to coincide with the start and stop of each gaze trial. Participants were not aware of the trial order or duration of gaze. This was due to previous studies in our lab showing anticipatory arousal (i.e., increase in SCL) for the eye contact trials before the trial started, which we wanted to mitigate as much as possible. Thus, participants were given the instruction to perform each “pose” before each gaze trial. The order of the gaze trials remained the same for each block but was counterbalanced across blocks. For example, the clear condition had the trial order of A (no-gaze), B (send), C (receive), and D (eye contact); the degraded condition had A (no-gaze), C (receive), D (eye contact), and B (send); and blocked condition had A (no-gaze), D (eye contact), C (receive), and B (send). The no-gaze trials were always first because they acted as a baseline measure of arousal for each condition, since electrodermal activity has been shown to steadily change (increase in some and decrease in others) over the course of the experimental session. In other words, we wanted to evaluate the base level of arousal for each participant at the beginning of each testing block, and then be able to compare that arousal level to the following key gaze trials within that block.

The experiment took approximately 45 min to complete. Upon completion, participants had the physiological equipment removed and were verbally debriefed.

#### Data Analysis

The skin conductance data was manually reviewed offline using the *Thought Technology* software *Physiology Suite* and any visible artefacts were removed (less than 1% of each participants data was removed). No high-pass or low-pass filters were needed. The data was then exported to Excel and imported into a custom Matlab program where it was epoched by participant, condition, and gaze trial. This program also baseline-corrected the data to 8-s before the start of each trial. We chose a longer baseline to include the electrodermal change from the start of the trial, which included the investigator giving the gaze instructions. Data were included even if participants demonstrated little change in skin conductance, but would have been removed if participants demonstrated a change too soon (0.1 μS within the first second) after the trial started, since this response would not have been elicited by the stimulus (Dawson et al., 2000). However, this did not occur in any of the data collected and therefore none was removed.

The data was analyzed in terms of both skin conductance responses (SCRs) and skin conductance levels (SCLs) in SPSS. SCRs were defined as the mean amplitude across the first 10 s of each gaze trial, while the SCLs were calculated as the average of the entire 60-s epoch. Mean SCRs and mean SCLs were submitted to a 2 (*Participant*: A or B) × 3 (*Condition*: clear, degraded, blocked) × 4 (*Gaze trial*: no-gaze, send-only, receive-only, send/receive) mixed Analysis of Variance (ANOVA) with the between-subject factor *Participant* and within-subject factors *Condition* and *Gaze Trial*, where sphericity was violated, a Greenhouse-Geiser correction was used.

### Results

Our hypothesis was that the arousal (SCR and SCL) in response to eye contact would be significantly higher in the natural condition (clear *sending* and *receiving* of gaze signals that significantly differs from other trials), lower for the sunglasses condition (degraded gaze signals, resembling more *send* or *receive* gaze trials), and lowest for the blindfolded condition (blocked gaze signals, resembling *no-gaze* trials).

#### Skin Conductance Response Analysis

**Figure 2:**
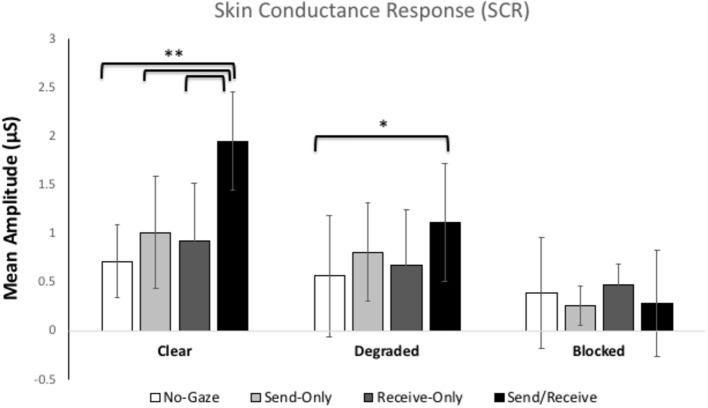
Mean skin conductance responses (SCRs) for each gaze trial (*no-gaze, send, receive, and send/receive or eye contact*) across the three Conditions (*clear, degraded, blocked*). The error bars represent the 95% confidence intervals. ** represents *p* < 0.001, * represents *p* < 0.01.

Figure 2 shows the mean skin conductance responses for each gaze trial in each condition. The ANOVA revealed significant main effects for Condition, *F*(2, 116) = 7.755, *p* = 0.001, *η*^2^ = 0.12 and Gaze Trial, *F*(3, 174) = 3.42, *p* = 0.05, *η*^2^ = 0.06. Most importantly, there was a significant interaction between Condition and Gaze Trial, *F*(6, 348) = 12.344, *p* < 0.001, *η*^2^ = 0.18. Pairwise Bonferroni comparisons showed a significant difference between the natural and blindfold conditions (*p* < 0.001), such that the natural condition elicited the overall highest SCR (*M* = 1.15 μS, SE = 0.14), while the blindfolded condition showed the lowest overall SCR (*M* = 0.353 μS, SE *=* 0.11). The ANOVA also revealed Pairwise Bonferroni comparisons showed a significant difference between eye contact and away trials, whereby the eye contact trial elicited the largest SCR (*M* = 1.12 μS, SE *=* 0.16) and the away trial showed the lowest SCR (*M* = 0.559 μS, SE *=* 0.15). There was no between-subjects difference in SCR between participants A and B.

#### Skin Conductance Level Analysis

**Figure 3:**
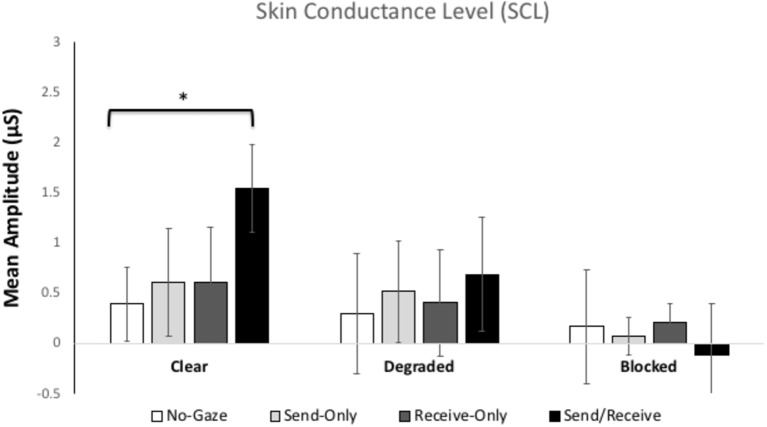
Mean skin conductance levels (SCLs) for each gaze trial (*no-gaze, send, receive, and send/receive or eye contact*) across the three Conditions (*clear, degraded, and blocked*). The error bars represent the 95% confidence intervals. * represents *p* < 0.001.

Figure 3 shows the mean skin conductance levels (average of the 60-s) for each gaze trial in each condition. The ANOVA revealed significant main effects of Condition, [*F*(2, 112) = 5.951, *p* = 0.01, *η*^2^ = 0.10] a marginally significant main effect of Gaze Trial, [*F*(3, 168) = 2.35, *p* = 0.07, *η*^2^ = 0.04]. Most importantly, there was a significant interaction between Condition and Gaze Trial, [*F*(6, 336) = 12.728, *p* < 0.001, *η*^2^ = 0.19]. There was no between-subjects difference in SCR between participants A and B.

Since the hypotheses were separate by condition, we decided to investigate the significant interaction by conducting separate one-way ANOVAs for each condition separately, as well as a one-way ANOVA for the eye contact trials (*send/receive*) across the three conditions. A Bonferroni correction was used for multiple comparisons (*alpha of p <* 0.012).

##### Clear Condition

In this condition, we predicted that eye contact (*send/receive trials*) would elicit significantly higher arousal responses compared to all of the other gaze trials (*send-only, receive-only,* or *no-gaze*). The ANOVA revealed a significant SCR difference across Gaze Trials [*F*(3, 174) = 10.547, *p* < 0.001, *η*^2^ = 0.15] and SCLs [*F*(3, 174) = 9.77, *p* < 0.001, *η*^2^ = 0.14]. Pairwise Bonferroni comparisons showed that eye contact trials elicited higher SCRs compared to away trials (*p* < 0.001), send trials (*p* = 0.015), and receive trials (*p* = 0.004). The SCL analysis mirrored that, with eye contact being significantly higher compared to away trials (*p* < 0.001), send trials (*p* = 0.01), and receive trials (*p* = 0.006). There were no between-subjects differences between participants A and B.

##### Degraded Condition

In this condition, eye gaze was degraded by sunglasses, and thus it might be hard for participants to send or receive gaze signals at the same time. As such, we predicted that eye contact would elicit a similar arousal response as the *send-only* and *receive-only* trials. Consistent with our prediction, the ANOVA revealed only a marginal SRC effect for Gaze Trial [*F*(3, 174) = 2.88, *p* = 0.065] and no SCL effect [*F*(3, 174) = 1.267, *p* = 0.287, *η*^2^ = 0.02]. Pairwise Bonferroni comparisons on the SCRs showed there was a significant difference only between the eye contract trials and away trials (*p* < 0.05). There were no between-subjects differences between participants A and B.

##### Blocked Condition

In this condition, eye gaze was blocked by a blindfold. Since eye contact cannot be made in this case, we predicted arousal to be the same across all Gaze Trials. As expected, the ANOVA did not reveal a significant SCR effect for Gaze Trial [*F*(3, 174) = 0.522, *p* = 0.668, *η*^2^ = 0.009] nor for SCLs [*F*(3, 168) = 0.701, *p* = 0.553, *η*^2^ = 0.01]. However, unlike the previous conditions, both SCRs [*F*(1, 58) = 4.838, *p* < 0.05] and SCLs [*F*(1, 56) = 3.98, *p* = 0.05] revealed a marginally significant difference between participant A and participant B, such that Participant B showed higher SCRs and SCLs (*M_SCR_* = 0.667 μS, *M_SCL_* = 0.297 μS) for all gaze trials compared to Participant A (*M_SCR_* = −0.105 μS, *M_SCL_* = −0.084 μS). This finding is likely due to Participant B being the one blindfolded and therefore subject of attention.

#### Eye Contact Across Conditions

**Figure 4:**
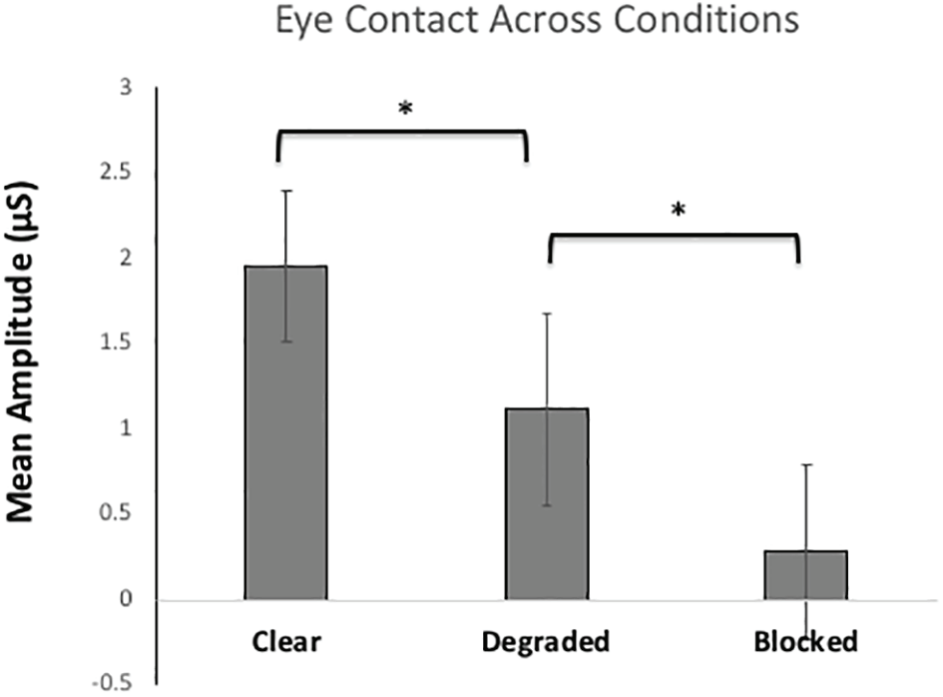
Mean skin conductance responses (SCRs) for the eye contact trials (*sending*/*receiving* gaze signals) for each of the three Conditions (*clear, degraded, blocked*). The error bars represent the 95% confidence intervals. * represents *p* < 0.001.

Figure 4 shows the mean skin conductance responses for eye contact trials in each condition. Here, we predicted that arousal elicited by eye contact would be modulated by the clarity of the gaze signals. As such, the clear condition should elicit the highest arousal gaze signals can be both sent and received clearly. However, once the gaze signals are degraded, there will be less arousal because there might only be one gaze signal (send or received) that is processed and when signals are blocked completely with no gaze signals involved, arousal should be lowest. The ANOVA compared SCRs for eye contact trials across each condition (*clear, degraded, and blocked*). The results showed a significant main effect of condition, *F*(2, 116) = 25.83, *p* < 0.001, *η*^2^ = 0.31. Pairwise Bonferroni comparisons showed significant differences between every condition, with the clear condition eliciting the highest arousal compared to degraded (*p* < 0.01) and blocked (*p* < 0.001). The degraded condition also showed significantly higher arousal than blocked (*p* < 0.01). There were no between-subjects differences between participants A and B.

#### Arousal as a Function of Time

**Figure 5:**
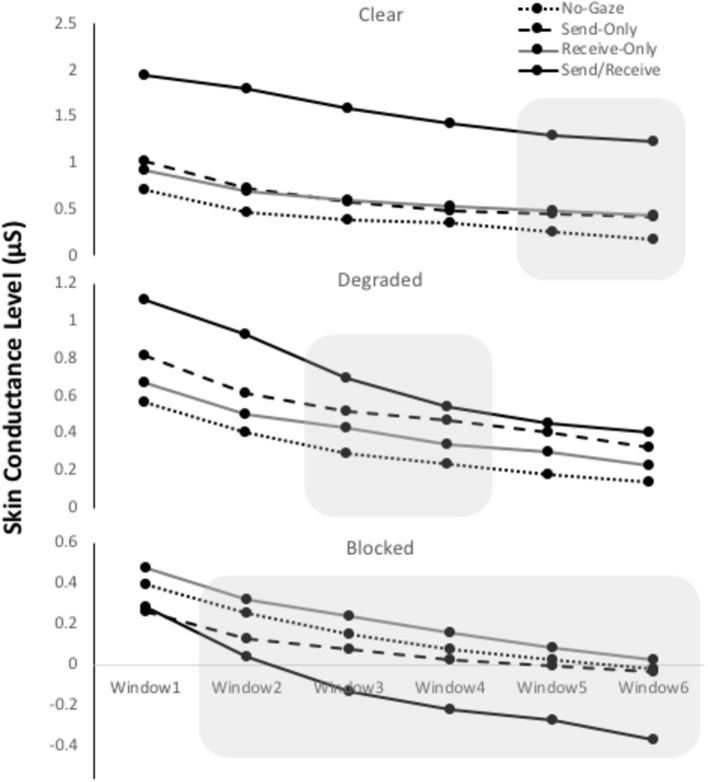
Mean skin conductance levels (SCLs) for each gaze trial (*No-Gaze, Send-Only, Receive-Only, and Send/receive*) for each of the three Conditions (*clear, degraded, and blocked*). The 1-min interval was epoched into six 10-s time windows. The shadow represents a significant difference found in the interaction between gaze trial and time (*p* < 0.001).

The mean skin conductance level for each participant was epoched into six time-windows of 10 s each. The data for participants A and B can be seen in Figure 5. The means for each epoch were submitted to a 2 (*Participant*: A or B) × 4 (*Gaze trial*: no-gaze, send-only, receive-only, send/receive) × 6 (*Time:* 1, 2, 3, 4, 5, 6) mixed Analysis of Variance (ANOVA) with the between-subject factor *Participant* and within-subject factors *Gaze Trial* and *Time*. A Bonferroni correction was used for multiple comparisons.

##### Clear Condition

The ANOVA revealed a significant main effect of Gaze Trial [*F*(3, 174) = 8.13, *p* < 0.001, *η*^2^ = 0.12], a main effect of Time [*F*(5, 290) = 53.22, *p* < 0.001, *η*^2^ = 0.48], and an interaction between Gaze Trial and Time [*F*(15, 870) = 3.19, *p* < 0.001, *η*^2^ = 0.05]. Within-subject contrasts of Gaze Trial showed that eye contact trials were significantly higher in SCL compared to the other trials, *F*(1, 58) = 17.43, *p* < 0.001, *η*^2^ = 0.23. Within-subject contrasts of Time showed that every time-window was significantly lower than the one before it (all *p*’*s* < 0.001) suggesting that SCL continually declined over 1 min. Within-subject contrasts involving the interaction showed a significant higher SCL between the eye contact trials and the rest during time-windows 5 (*p* < 0.005) and 6 (*p* < 0.02), suggesting that the SCL during eye contact stayed high during 1 min. There was no difference between participants A and B.

##### Degraded Condition

The ANOVA revealed a significant main effect of Time [*F*(5, 290) = 56.37, *p* < 0.001, *η*^2^ = 0.49], and an interaction between Gaze Trial and Time [*F*(15, 870) = 3.97, *p* < 0.001, *η*^2^ = 0.06], but no main effect of Gaze Trial. Within-subject contrasts of Time showed that every time-window was significantly lower than the one before it (all *p’s* < 0.001) suggesting that SCL continually declined over 1 min. Within-subject contrasts involving the interaction showed a significant SCL difference for eye contact trials from the rest of the gaze trials until time-window 4 (*p* < 0.005). This suggests that the SCL for eye contact declined to the level of the other gaze trials after time about 30 s. There was no difference between participants A and B.

##### Blocked Condition

The ANOVA revealed a significant main effect of Time [*F*(5, 290) = 60.75, *p* < 0.001, *η*^2^ = 0.52], and an interaction between Gaze Trial and Time [*F*(15, 870) = 4.95, *p* < 0.001, *η*^2^ = 0.08], but no main effect of Gaze Trial. Within-subject contrasts of Time showed that every time-window was significantly lower than the one before it (all *p* < 0.001) suggesting that SCL continually declined over 1 min. Within-subject contrasts involving the interaction showed a significant SCL difference from the rest of the gaze trials during time-windows 2, (*p* < 0.005), 3 (*p* < 0.001), 4 (*p* < 0.001), and 6 (*p* < 0.002). This interaction suggests that eye contact trials began a significant decline more than the other gaze trials. There was no difference between participants A and B.

## Experiment 2

It could be argued that the heightened autonomic nervous system arousal observed when participants made eye contact was *not* due to the sending and receiving of gaze information (and an increased opportunity for social interaction) but instead a consequence of viewing another person’s eyes. The eyes are salient attention-capturing stimuli and this alone may be responsible for the increase in SCRs. Moreover, one could argue that the blocked and degraded conditions elicited lower SCRs not because they hindered the participants’ ability to successfully send and receive gaze information, but instead because they degraded the visibility (and thus the saliency) of the eyes. To eliminate the arousal associated with just seeing the eyes, we conducted a *post hoc* experiment where participants performed the gaze trials with themselves through a mirror. Since there would be no need to send or receive gaze signals with oneself, we assumed that any potential for social interaction would be eliminated. Yet in this scenario, participants are still making eye contact with a pair of real eyes and thus, the saliency of the eyes remains constant. If making eye contact with oneself in the mirror is less arousing than making eye contact with another person, then that would demonstrate there is something beyond the saliency of the eyes that is driving the enhanced arousal seen in Experiment 1. We hypothesized there to be no significant difference in physiological response between any of the gaze trials because there would be no need to send and/or receive signals when making eye contact with oneself. If our predictions are supported, the results would more strongly speak to the notion that the heightened arousal found in Experiment 1 is due to both individuals sending *and* receiving gaze information, which contributes to the potential for further social interaction.

### Method

#### Participants

Eleven MacEwan University undergraduates were recruited to participate (2 males, 8 females; average age = 21 years old). All were right-handed with normal or corrected-to-normal vision and hearing. Data from one participant was not included in the analysis due to instructions not being followed (they moved their whole body during the gaze trials instead of just their head as instructed).

#### Materials and Procedure

Participants were situated in front of a 27″ iMac computer where a picture was taken of their averted face (i.e., their profile) using Photo Booth. Participants were then fitted with physiological monitoring equipment (*Thought Technology, Inc.*) whereby two Ag/AgCl electrodes were attached to the palmar surface and of the distal phalanxes of their ring and index finger of their left hand. Their skin conductance level (SCL) was collected at a sampling rate of 256 samples per second. Participants engaged in three gaze trials that attempted to replicate those in Experiment 1: (1) looked at the wall (*no-gaze trials*), (2) looked at their own averted face on the computer screen (*send-only trials*), and (3) made eye contact with themselves in a mirror (*send/receive or eye contact trials*). Each trial lasted for 1 min and was signaled by the experimenter to begin and end. Participants remained relatively still throughout the experiment to prevent movement artifacts, with 1–2 min breaks between trials to allow for movement if needed. Following the three gaze trials, participants were then detached from the physiological equipment and filled out a brief questionnaire that assessed the degree to which looking at themselves in the mirror provoked negative (e.g. disgust, awkward) or positive (e.g. attraction, content) emotions. The questionnaire was used to ascertain whether any arousal observed was associated with making eye contact with oneself in the mirror or whether it could have been attributed to the emotions elicited by looking at oneself in the mirror. The experiment took approximately 15 min to complete.

### Results

Similar to Experiment 1, data was pre-processed and artifact-checked for each gaze trial with the removal of an 8-s anticipatory phase during the instructions. Mean skin conductance responses (SCRs) were calculated by averaging the amplitude across the first 10 s while the skin conductance levels (SCLs) were calculated as the average amplitude of the entire 1-min epoch. Mean SCRs and SCLs were baseline-corrected to 1 s before anticipatory began. Data was included even if participants demonstrated little change in skin conductance across the trials. The average SCRs across the gaze trials can be seen in Figure 5.

Mean SCRs and SCLs were submitted to a one-way Analysis of Variance (ANOVA) with the within-subjects factor Gaze Trial (*no-gaze, send-only, and send/receive*). As predicted, the ANOVAs revealed no significant main effect across Gaze Trials for SCRs [*F*(2, 18) = 2.117, *p* = *n.s.*] and SCLs [*F*(2, 18) = 1.656, *p = n.s.*], such that making eye contact with oneself was no more arousing than the send or away trials. It should be noted however that the sample size is small and observed power was low (0.3). The small sample also lends itself to greater variability which may impact the ANOVA results. However, the raw data for each participant can be seen in Table 1, showing that only two participants had even a hint of the trend toward greater SCR for eye contact than the other trials (represented by *). All other participants showed either no change in SCR over each trial or in the opposite direction expected.

**Table 1:** Individual mean skin conductance responses (SCRs) for the mirror condition (Experiment 2).

No-gaze	Sent-only	Eye contact	Eye contact – No-gaze
0.297	0.273	0.394	0.096
0.684	1.956	3.296	2.611*
0.232	0.601	0.715	0.482
0.016	0.164	1.091	1.074*
0.185	0.249	0.206	0.020
1.372	1.894	1.354	−0.018
0.273	0.307	0.205	−0.069
0.086	−0.001	0.043	−0.044
1.666	1.411	1.421	−0.246
0.889	0.771	1.133	0.244

The last column represents the skin conductance change between the no-gaze and eye contact trials. Only two participants showed even a hint of the eye contact trials eliciting higher SCRs, indicated by the *.

### Discussion

The purpose of this study was two-fold: first to demonstrate that live eye contact between two strangers can elicit heightened autonomic nervous system arousal due to the sending *and* receiving of gaze signals; and second, to evaluate whether arousal can be modulated by the clarity of gaze information. For example, can the eyes elicit arousal even if the information received from them is unclear? To test this, we monitored participants’ skin conductance level while they maintained gaze for 1 min in a clear condition, degraded condition (sunglasses), and blocked condition (blindfolded). Hence, our hypotheses centered around how eye contact (*send/receive trials*) would activate nervous system arousal in each other the three clarity conditions. Thus, our predictions were three-fold: (1) we predicted that arousal will be enhanced the most when participants make eye contact over any other gaze trial (*send-only, receive-only, or no-gaze*) because participants will be *sending* and *receiving* gaze signals; (2) we predicted that arousal during degraded eye contact trials (with sunglasses) will be similar to the send-only and receive-only trials, where only one signal is being monitored/processed; and (3) lastly, we predicted that the arousal for the blindfolded eye contact trials will not differ from the no-gaze trials, since no gaze signals are being sent or received.

There are two unique characteristics of our design that warrant a second mention. One is that this might be the first study to observe and measure autonomic nervous system arousal of two live participants engaged in different gaze conditions—typically research has measured a participant response to a live confederate, or response of participant to a static image of direct gaze. As such, our design has strong ecological validity. Second, our design required participants to maintain the gaze (e.g., eye contact) for 60-s intervals—a time period that is much longer in duration than what is typically used in the eye gaze literature (~3–5 s; Helminen et al., 2001). This prolonged time period allows the flexibility to analyze the skin conductance response (SCR) when eye contact is made (i.e., initial gaze response), as well as the change in skin conductance level (SCL) over time due to continuous sending and receiving of gaze information (i.e., gaze communication). Thus, we afforded the opportunity to evaluate arousal to gaze as a function of time, giving more insight into a complex process.

Our main finding was that arousal was highest when participants made eye contact and when the gaze signals were clearly sent and received simultaneously. We believe that the arousal enhancement was due to the combined, simultaneous tasks of monitoring one’s own gaze signals and interpreting the gaze signals of others. This finding is consistent with the recent eye tracking data from Hessels et al. (2019) who simultaneously recorded gaze from two interacting participants. They reported that gaze depends on the sub-task, such as speaking versus listening. The results showed that people will monitor gaze for cues about when speaking will commence. This gaze monitoring is analogous to what we mean by *receiving* gaze signals. For example, while one person is sending the gaze signal regarding speaking, the other is sending out signals regarding listening. Interestingly, eye contact trials in the clear condition were the only trials that significantly differed in skin conductance level (SCL) from the other gaze trials. Thus, not only did eye contact initially elevate arousal, but it stayed elevated for the 1-min epoch (see Figure 5). In terms of sending and receiving gaze signals, this finding suggests that we keep processing and interpreting gaze information continually while eye contact is made. In many animals, prolonged gaze is typically associated with aggression or intimidation (Emery, 2000; Skuse, 2003). In humans, sustained eye contact has been linked to expressing control/dominance or love/inclusion (Argyle et al., 1974; Kellerman et al., 1989; Hall et al., 2005; for a review, see Hietanen, 2018). Although there was no reason for participants in this study to convey emotional feelings, especially when they were clearly instructed to remain neutral, it is possible that some emotional processing was going on between the eyes. Measuring the affective relationship with arousal level and gaze duration in humans is an avenue for future research.

With regards to signal clarity, we found that as the gaze signals became degraded, arousal was diminished. That is, when participants made eye contact with someone wearing sunglasses (where the eyes can be seen but the signals cannot be received), arousal was significantly lower than in the clear condition. Indeed, the arousal associated with eye contact in the degraded condition was on the same level (not significantly different) from the arousal elicited by the send-only or receive-only conditions. This finding suggests that when gaze signals are degraded, only one process (either sending *or* receiving occurs). For instance, if you are the person wearing the sunglasses, then you do not need to self-monitor your own gaze because those signals cannot be clearly received by an observer. Similarly, if you are the person looking at someone wearing sunglasses, you can send gaze signals and need to self-monitor, but you cannot receive the signals clearly and therefore do not need to process or interpret those signals. Thus, arousal in this case might only be associated with either self-monitoring one’s own gaze signals, *or* interpreting others gaze signals, but not both. This finding is not consistent with Myllyneva and Hietanen (2015) who found an increase in arousal (skin conductance response) for eye contact compared to averted gaze when the gaze signals were clear and when they were degraded by sunglasses (but not when blocked). However, depending on the tint of the sunglasses and the distance of the model, gaze signals might have been clear enough to interpret. In our study here, sunglasses were tinted to the degree that they eyes were noticeable, but eye movements were not able to be tracked. Given the limited number of trials in our study, we might have found an effect similar to Myllyneva and Hietanen (2015) had we tested additional participants.

We did find a similar result as Myllyneva and Hietanen (2015) when the gaze signals were blocked in the blindfold condition, where no gaze signals could be sent or received. Myllyneva and Hietanen (2015) found that eye contact with a model wearing opaque glasses was the same as the model averted. We found the same result, such that the arousal in the eye contact trials was not significantly different from the *no-gaze* trials. However, planned comparisons did show an interesting effect of Participant A (not manipulated) compared to B (wore the blindfold). Participant B showed higher SCRs and SCLs for all gaze trials compared to Participant A. These results could have been due to participant B “believing” that they were the center of someone’s attention, as described in Myllyneva and Hietanen (2015, 2016). Myllyneva and Hietanen suggested that even the thought that someone was looking at you could be enough to generate an arousal response associated with an increase in self-awareness. Perhaps that was the experience of Participant B, but more research is needed to directly test these speculations.

**Figure 6:**
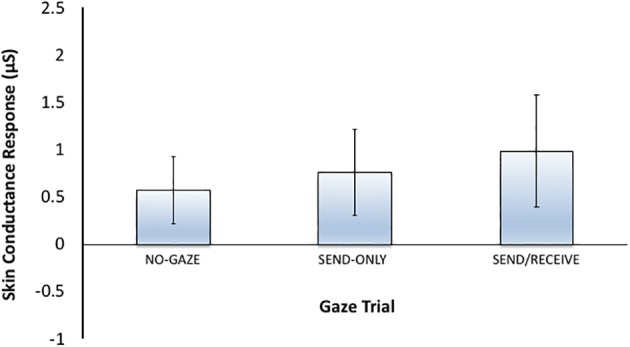
Mean skin conductance responses (SCRs) for each gaze trial (*No-Gaze, Sent-Only, and Send/Receive or eye contact*) for Experiment 2 (Mirror control condition). The error bars represent the 95% confidence intervals.

In Experiment 2, the heightened arousal found during eye contact in the clear condition in Experiment 1 was not observed when participants locked eyes with themselves in a mirror. Instead, as seen in Figure 6, participants SCRs when looking in the mirror looked very similar in magnitude to the SCRs observed in the sunglasses condition when the gaze signal was degraded. That said, there is one similarity between the mirror and sunglasses conditions–the degree to which the eyes could be perceived. In both cases, the eyes were visible (to a degree) and looking back. While in the sunglasses condition the potential to interact with another person was still available, in the mirror condition this potential was nonexistent. However, signals could either be sent or received in the sunglasses condition, whereas sending/receiving gaze signals with oneself is redundant. Thus, the arousal observed in the eye contact trials during the sunglasses condition could be simply due to the saliency of the eyes perceived through the sunglasses, or due to either sending gaze or receiving gaze. Given the small sample size (*n* = 10) and variability in the data, we are cautious to make any strong conclusions from this *post hoc* control experiment. However, future research could examine eye gaze with oneself in a mirror more thoroughly to see how gaze is interpreted and modulated. Perhaps gazing at one’s own reflection would increase self-awareness and cause heightened arousal under certain conditions.

Altogether these findings support the notion that there is something special about making eye contact with a live person and the arousal observed is likely the result of both sending and receiving gaze information. Research has proposed that making eye contact with a live person opens the door for social interaction (Laidlaw et al., 2011), and is likely that initial message that is responsible for the immediate boost in skin conductance response. In other words, making eye contact with another person might at first be processed as an approach signal to socially interact. One theory put forth by Hietanen et al. (2008) is that direct gaze may signal to the receiver that there is an intent to approach, while averted gaze may signal the intent to avoid. Hietanen et al. (2008) found that eye contact with a confederate resulted in heightened activity in the left frontal cortex and enhanced arousal, both of which are responses associated with the motivational tendency to approach. The same study found that perception of an averted face elicited greater activity in the right frontal cortex, indicative of the motivational tendency to avoid (Hietanen et al., 2008). Congruent with these findings were participants’ subjective reports of higher arousal in the eye contact condition and the increase in approach motivation compared to the averted condition (Hietanen et al., 2008). However, the differential gaze effect was only significant when participants made eye contact with a live confederate but not when they made eye contact with an image of the confederate (Hietanen et al., 2008). Since the effect was only observed with a live confederate, this provides evidence that we only use eye contact to signal approach if there is someone to approach and interact with.

This approach/avoidance theory is not new and was first observed by Cary (1978) who video-taped conditions where a naïve participant was seated in a waiting room while another person entered the room. Social interaction (brief or continuous) was more likely to be observed if mutual gaze (or eye contact) between dyads occurred once the participant entered the room. Continuous conversation was even more likely to occur if mutual gaze occurred a second time upon entry. Alternatively, Cary (1978) found that the absence of mutual gaze upon entry predicted little to no social interaction between dyads. While this was an observational study, it does support the notion that it is the mutual exchange (i.e., sending and receiving) of approach signals that prompts the potential for social interaction, and a mutual exchange of avoidance signals (if one or both individuals avert their gaze from each other) decreases the potential for social interaction.

The intention to approach someone and socialize would be suitable for activating the nervous system within the first few seconds (SCR), but we found here that eye contact maintains the heightened arousal response for the 60-s duration (SCL). What signals could be sent and received on a continuous basis as to sustain an elevated level of arousal? Some researchers have suggested the existence of “a social brain network” (Johnson et al., 2005; Adolphs, 2009) specialized in processing social information that is modulated by eye contact (Senju and Johnson, 2009). In line with this, Conty et al. (2016) put forth the Watching Eyes model, which suggests that gaze is processed in two stages: first, the eyes capture attention and we processes whether the eyes are looking at us or not, and then the second stage activates internal processing generated by eye contact that self-referential in nature and can be associated with pro-social behaviors and positive appraisals of others. This second stage is consistent with Hietanen and colleagues, who have shown that self-referential processing could occur when we just “believe” someone is looking at us, regardless of seeing the eyes (Myllyneva and Hietanen, 2015; although see Myllyneva and Hietanen, 2016). Also in line with this, Cavallo et al. (2015) found that live gaze (direct and averted) elicited significant activation in brain areas involved in inferring mental states during social interactions (anterior rostral medial prefrontal cortex or arMPFC) as well as language comprehension and production [inferior frontal gyrus (IFG), premotor cortex, and supplementary motor area]. Further analysis showed that there was an increase in connectivity between the IFG and arMPFC during live eye contact, suggesting that it triggers a network of brain regions involved in the detection of communicative intentions and language. Similarly, a desynchronization of alpha-band activity was observed when infants looked at an object together with an adult during a social interaction involving eye contact (Hoehl et al., 2014). No such effect was observed when infants and adults were not engaged in eye contact. Thus, it is likely that the *received* gaze signals that we refer to in our study are analogous to these mentalizing processes, such that we are continually interpreting others gaze to understand their intentions, desires, beliefs, and knowledge (Conty et al., 2016). It is also likely that the *sent* gaze signals are related more to an increase is self-awareness (Conty et al., 2016), and an overall heightened attention to monitor what gaze signals we want to be public. For example, if we are lying, we might conceal gaze signals to not show the truth. Or if we are angry or sad, we might avoid gaze with others as to hide our feelings that could be communicated unwillingly through eye contact.

Altogether, our findings contribute to the previous literature by showing that arousal is elicited most strongly in the first 10 s during eye gaze if it is clear that the eyes are looking back (consistent with Watching Eyes model stage 1), and then the individual maintains a high level of arousal for the duration of the eye gaze, likely in response to mentalizing processes (self-reference, self-monitoring, communication, etc.) that occur thereafter (consistent with Watching Eyes model stage 2). This arousal pattern elicited in the clear condition, but once the gaze signals could not be interpreted clearly, like when someone wears sunglasses in the degraded condition, arousal dropped back down to baseline levels after about 30 s. Thus, we believe that the arousal level sustained over the entire minute was not due to the observer just self-managing (Silver and Shaw, 2018), but also due to the online and consistent perceiving and interpreting other’s gaze for information related to their mental state, emotion, intention, attention, etc. (Baron-Cohen, 1995).

## Limitations

Measuring the behavior of two live participants, while rich in data, is not without its limitations. For instance, participants who wore the sunglasses (participant B) verbally mentioned that they were uncertain of the extent to which the sunglasses disguised their eyes to the other participant. Based on informal conversations, the majority of participants who wore the sunglasses assumed their eyes were quite visible and thus, they would believe they could send gaze information to the other person. However, this should have been consistent with every participant either believing that their eyes were visible or not. Another potential limitation was the within-subjects design, such that participants took part in all three conditions—clear, degraded, and blocked. While the order of conditions was counterbalanced, previous (unpublished) research in our lab has shown that participants habituate to eye contact over time and show less and less arousal with repeated exposure. Thus, our data may have been stronger if we had enough participants to analyze the data as a between-subjects design. Lastly, in the blindfold condition it was assumed that the blindfold would prevent all gaze signals from being sent and received between dyads because participant B’s eyes were entirely concealed. Since both participants were expected to have no ability to send or receive gaze information, no differences in the SCR between partners were expected to emerge in the eye contact trials. As mentioned in the results, there was a difference between participants arousal levels within the blindfold condition, such that participant B (wearing the blindfold) showed significantly higher arousal across all gaze trials. One possibility is that simply being blindfolded increased arousal because of the knowledge of being the focus of someone’s attention. Thus, being the object of someone’s attention could have been driving the arousal response.

## Conclusion

The current study demonstrated that arousal from eye contact is associated with the sending *and* receiving of gaze signals, and as the ability to exchange gaze signals decreases (by degrading the visibility of the eyes with sunglasses or a blindfold), so does arousal and the possibility for social interaction. We also tried to rule out the argument that arousal from eye contact is due to the saliency of the eyes by demonstrating no arousal enhancement when participants made eye contact with themselves in a mirror.

These findings could have implications for individuals who wear sunglasses in our everyday life. From the sender’s point of view, it might be helpful to know that while wearing sunglasses during a social interaction (e.g., interview, business deal, romantic date, etc.), gaze information might not be communicated clearly, if at all. This lack of gaze information could hinder the communicative process by decreasing arousal and in turn reduce attention to, interest in, and excitement for what is being said. From the receiver’s point of view, it might be helpful to know that if someone is wearing sunglasses they might be doing so because they are not open to engaging in social interaction. That is, they might be trying to conceal their eyes in order to reduce the gaze signals of approach. On the other hand, if you wish to engage in riveting social interaction, then perhaps sunglasses should be avoided.

## Ethics Statement

All procedures were approved by the MacEwan University Research Ethics Board and participants gave informed consent.

## Author Contributions

MJ was the lead investigator for all of the projects reported and was primarily responsible for design conception, data management and analysis, and report composition. RB was involved in data collection, management, and organization.

### Conflict of Interest Statement

The authors declare that the research was conducted in the absence of any commercial or financial relationships that could be construed as a potential conflict of interest.
